# Prevalence of Virulence Determinants and Antimicrobial Resistance among Commensal *Escherichia coli* Derived from Dairy and Beef Cattle

**DOI:** 10.3390/ijerph120100970

**Published:** 2015-01-19

**Authors:** Ewa Bok, Justyna Mazurek, Michał Stosik, Magdalena Wojciech, Katarzyna Baldy-Chudzik

**Affiliations:** 1 Department of Molecular Biology, Faculty of Biological Sciences, University of Zielona Góra, Monte Cassino St. 21b, 65-561 Zielona Góra, Poland; E-Mails: j.mazurek@wnb.uz.zgora.pl (J.M.); m.stosik@wnb.uz.zgora.pl (M.S.); k.baldy-chudzik@wnb.uz.zgora.pl (K.B.-C.); 2 Department of Mathematical Statistics and Econometrics, Faculty of Mathematics, Computer Science and Econometrics, University of Zielona Góra, Prof. Z. Szafrana St. 4a, 65-516 Zielona Góra, Poland; E-Mail: m.wojciech@wmie.uz.zgora.pl

**Keywords:** commensal *Escherichia coli*, dairy cattle, beef cattle, phylogenetic grouping, virulence factors, antimicrobial resistance

## Abstract

Cattle is a reservoir of potentially pathogenic *E. coli*, bacteria that can represent a significant threat to public health, hence it is crucial to monitor the prevalence of the genetic determinants of virulence and antimicrobial resistance among the *E. coli* population. The aim of this study was the analysis of the phylogenetic structure, distribution of virulence factors (VFs) and prevalence of antimicrobial resistance among *E. coli* isolated from two groups of healthy cattle: 50 cows housed in the conventional barn (147 isolates) and 42 cows living on the ecological pasture (118 isolates). The phylogenetic analysis, identification of VFs and antimicrobial resistance genes were based on either multiplex or simplex PCR. The antimicrobial susceptibilities of *E. coli* were examined using the broth microdilution method. Two statistical approaches were used to analyse the results obtained for two groups of cattle. The relations between the dependent (VFs profiles, antibiotics) and the independent variables were described using the two models. The mixed logit model was used to characterise the prevalence of the analysed factors in the sets of isolates. The univariate logistic regression model was used to characterise the prevalence of these factors in particular animals. Given each model, the odds ratio (OR) and the 95% confidence interval for the population were estimated. The phylogroup B1 was predominant among isolates from beef cattle, while the phylogroups A, B1 and D occurred with equal frequency among isolates from dairy cattle. The frequency of VFs-positive isolates was significantly higher among isolates from beef cattle. *E. coli* from dairy cattle revealed significantly higher resistance to antibiotics. Some of the tested resistance genes were present among isolates from dairy cattle. Our study showed that the habitat and diet may affect the genetic diversity of commensal *E. coli* in the cattle. The results suggest that the ecological pasture habitat is related to the increased spreading rate of the VFs, while the barn habitat is characterised by the higher levels of antimicrobial resistance among *E. coli*.

## 1. Introduction

Commensal *Escherichia coli* plays a dynamic role in the ecology of intestinal tract, analyses of commensal population of *E. coli* derived from cattle is important for ecological and medical reasons, it allows to understand the role of commensals in transmission of virulence factors and antimicrobial resistance. The *E. coli* genome exhibits a high degree of heterogeneity therefore this bacteria can be a commensal organism, but on the other hand it can be also a dangerous pathogen causing intestinal or extraintestinal infections. Pathogenic potential of *E. coli* depends on the ability to the acquisition of mobile genetic elements carrying different virulence factors (VFs) [1,2]. Shiga toxin-producing *E. coli* (STEC) are one of the most important foodborne pathogens essential for public health, responsible for serious outbreaks, hemorrhagic colitis, and the hemolytic-uremic syndrome (HUS), the leading cause of acute renal failure [3,4]. Cattle is a natural reservoir of STEC and the transmission of this pathogen to humans can occur through the direct contact with infected animals, foods of animal origin such as meat or unpasteurized milk or via water contamination [4]. 

Nowadays in European Union the use of antibiotics is permitted only as therapeutic agents [5], but earlier antimicrobial agents had been used in animal production for therapeutic and growth promoting purposes. Many studies have demonstrated that antimicrobial resistance among *E. coli* derived from healthy food-producing animals is still common [6], therefore commensal *E. coli* can be used as an indicator organism to evaluate the prevalence of antimicrobial resistance. The presence of virulence factors can be closely connected with antimicrobial resistance among commensal *E. coli* isolated from cattle, thus it is of major public health significance related to risk of introducing these bacteria to the food chain [7,8]. 

Still little is known about differences between risks of food contamination by food-born and antimicrobial resistant pathogens connected with organic or conventional cattle production systems. Consumers nowadays prefer organic and natural products considering them to be safer, but the question arises if the organic cattle production system is really safe? There are only a few studies, concerning influence of this production system on prevalence of *E. coli* with pathogenic potential and antimicrobial resistance, available. The monitoring of the production system is important to human and animal health therefore further evaluation is necessary. The aim of this study was to evaluate the influence of two different habitats—the conventional barn and the ecological pasture on the phylogenetic structure, the distribution of virulence factors and prevalence of antimicrobial resistance among *E. coli* isolated from healthy cattle. 

## 2. Experimental Section

### 2.1. Bacterial Isolates Collection

Two cattle farms located in western Poland were selected for the purpose of this study. The first one represented a conventional dairy farm, where the cows have been kept in a free stall barn without pasture access. The second represented an organic farm with the ecological production management system; in this case beef cattle have been kept mainly on the ecological pasture. The diet of dairy cows consisted of corn silage, alfalfa and grass silage, beet bagasse and forage. The diet of beef cattle consisted of grass and straw. The antimicrobial agents were administered for therapeutic purposes only and cows were not treated with antibiotics within the three-month period prior to sampling. Animals in groups were housed together for at least one year. The group of dairy cattle lived in one barn and consisted of 50 adult individuals between the ages 5–8 years. The herd of beef cattle lived on one pasture and contained 42 adult individuals in the age 6–8 years. There were no calves in the tested groups. Freshly voided faecal samples were aseptically transferred into 100-mL polystyrene sterile containers. A total of 92 samples were taken throughout the study, 50 from all dairy cows in the group and 42 from all the cows in the beef cattle group. The tubes were transported to the laboratory on ice and processed the same day. The faecal samples were inoculated on m-FC agar (a medium to detect and enumerate faecal coliforms (FC)) and incubated at 44 °C for 24 h. The blue colonies were subcultured on the MacConkey’s agar and incubated at 37 °C for 24 h. Lactose-positive isolates were subjected to standard biochemical IMVC (indole, methyl-red-Voges-Proskauer, citrate) tests for identification. *E. coli* isolates were stored frozen as a glycerol stock, at −80 °C. DNA extraction was carried out using thermal cell lysis method; 1.5–3 µL of boiled bacterial supernatant was used as a template in PCR reactions. *E. coli* isolates were genotyped using DNA rep-PCR fingerprinting method with BOX-A1R primer as described earlier [9] to evaluate the genetic similarity. Based on the similarity of BOX-PCR genomic patterns, with the use of unweighted pair group method with arithmetic averages (UPGMA) and the Jaccard similarity coefficient, the non-identical isolates were determined. In total 265 unique *E. coli* isolates were identified, from one to three per one animal, 147 isolated from 50 dairy cows housed in the barn and 118 from 42 beef cattle living on the pasture. 

### 2.2. Phylogenetic Analysis

Isolates were assigned to the four major phylogenetic group by the method described previously [10], which is a rapid phylogenetic grouping technique based on triplex PCR of the *chuA* and *yjaA* genes and the DNA fragment TspE4.C2.

### 2.3. Virulence Factors Identification

PCR was used to screen isolates for the presence of 17 virulence factors. The genes examined included 13 intestinal (*stx1*, *stx2*, *escV*, *ehxA*, *bfpB*, *estI*, *estII*, *eltA*, *faeG*, *fanC*, *fasA*, *fedA*, *f41*) and four extraintestinal (*hlyA*, *fimH*, *papA*, *sfaS*) virulence determinants. PCR reactions were carried out using previously published primers and conditions [11,12]. The PCR amplification mixture in a volume of 25 µL contained: buffer solution (Thermo Scientific, Waltham, MA, USA); 2.5 mM MgCl_2_ (Promega, Madison, WI, USA); 0.5 mM of each dNTP (Promega); 0.2 µM of each primer (IDT, Coralville, IA, USA); 1 U of DyNAzyme II polymerase (Thermo Scientific) and 3 µL of DNA template. All PCR reactions included a negative control containing no DNA template and a positive control, containing DNA template from an *E. coli* isolates known to carry the identified gene. 

### 2.4. Analysis of Antimicrobial Susceptibility

The antimicrobial susceptibilities (phenotypes) of *E*. *coli* isolates to 13 antimicrobial agents were examined by the determination of minimum inhibitory concentration (MIC) using the broth microdilution method, following the Clinical and Laboratory Standards Institute (CLSI), Guideline [13]. Isolates were classified as susceptible or resistant based on epidemiological cut-off values issued by the European Committee on Antimicrobial Susceptibility Testing (EUCAST; http://www.escmid.org). The antibiotics cut-off values (mg/L) tested in this study were as follows: ampicillin (8), cefuroxime (8), ceftazidime (0.5), streptomycin (16), gentamicin (2), neomycin (8), tetracycline (8), doxycycline (8), sulphamethoxazole (256), trimethoprim (2) chloramphenicol (16), nalidixic acid (16), norfloxacin (0.25). *E. coli* strain ATCC 25922 was used as quality control for MIC assays. Isolates with an MIC above the cut-offs were considered as resistant, otherwise as susceptible. Multi-drug resistance (MDR) was defined as the non-susceptible profile to ≥1 agent in ≥3 antimicrobial categories.

### 2.5. Antimicrobial Resistance Genes Identification 

PCR to detect 11 resistance genes was performed on the isolates, according to their susceptibility patterns. The following resistance genes were analysed: *bla*_TEM_, *bla*_SHV_, *bla*_CTX-M_, *strA*/*strB*, *aadA1*, *tetA*, *tetB*, *tetC*, *sul1*, *sul2*, *dfrA1* and *dfrA7/dfrA17*. PCR reactions were carried out using previously published primers and conditions [14,15,16]. The PCR amplification mixture was prepared in a volume 25 µL as described above.

### 2.6. Statistical Analysis

Statistical analysis was performed using the statistical software package R distributed under an open-source license [17]. The presence of the VFs profiles and antimicrobial resistance were categorized as yes—“1” and no—“0”. The habitat of a given animal group (“cattle barn” and “cattle pasture”) were defined in the model as the independent variables. In each case “Cattle barn” was chosen to be the reference category. 

The relations between the dependent (VFs profiles, antibiotics) and the independent variables were described using two approaches: the mixed logit model for the set of isolates and the univariate logistic regression model for the particular individuals in the analysed set of animals. In the second approach each individual with at least one strain positive for virulence or resistance gene was considered as an individual with *E.* *coli*—positive for these factors. The mixed logit model approach takes into account the correlated nature of the outcomes. This method allows modelling the outcomes with dichotomous responses which include the measurements repeated on the same subject. Given each model, the odds ratio (OR) and the 95% confidence interval for the population were estimated. The association is deemed statistically significant at the 0.05 level, if the confidence interval does not contain 1.0. The differences in proportions between the analysed groups were compared using Pearson’s chi-squared test or Fisher’s exact test, depending on the context. In the case of all the statistical tests, the significance level was defined at 0.05.

## 3. Results 

### 3.1. Phylogenetic Grouping

**Figure 1:**
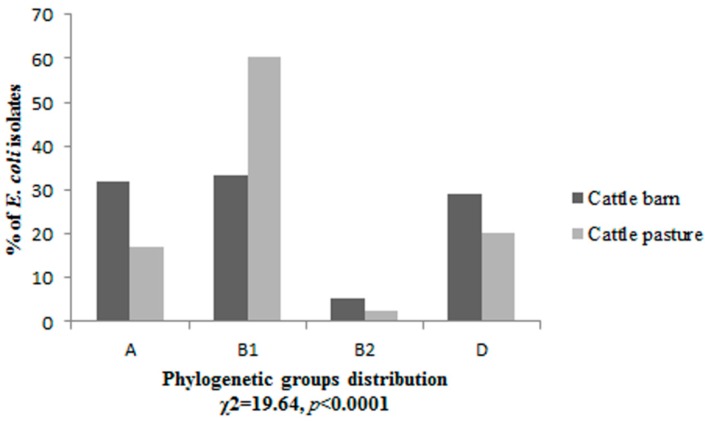
The phylogenetic structure of *E. coli* isolates derived from two groups of cattle housing in the barn and living in the pasture.

The four phylogenetic groups were not uniformly distributed (*p* < 0.0001) among isolates from dairy and beef cattle. *E. coli* isolates derived from cattle housing in the barn belonged to three phylogenetic groups A, B1 and D in almost equal amounts 32%, 33.3% and 29.3% respectively. Only 5.4% of isolates were classified as phylogenetic group B2. The majority of isolates 60.2% derived from cattle living on the pasture belonged to phylogenetic group B1, followed by 20.3% in group D, 17% in group A and 2.5% in B2 (Figure 1).

### 3.2. Prevalence of Virulence Factors 

Five intestinal virulence determinants: *stx1*, *stx2*, *escV*, *ehxA*, *estI* and two extra-intestinal: *hlyA*, *fimH* were detected among the analysed *E. coli* isolates. Genes *bfpB*, *estII*, *eltA*, *faeG*, *fanC*, *fasA*, *fedA*, *f41*, *papA*, *sfaS* were not found in any of the isolates. The *fimH* gene was present in all tested isolates, other reports also indicated current prevalence of this gene both among pathogenic and commensal *E. coli* [18,19] therefore, in the further analysis the *fimH* gene was not considered as a virulence factor. Five and eight different virulence profiles were observed among *E. coli* isolates derived from dairy and beef cattle respectively (Table 1A,B). Significantly more isolates derived from beef cattle (21.2%) than from dairy cows (6.1%) carried VFs (Table 1A). The comparison of frequency of STEC isolates also showed statistically significant differences, 12.7% isolates from beef cattle and 3.4% from dairy cows indicated the presence of STEC determinants (Table 1A). The results have shown that among both groups of cattle housing in the barn and living on the pasture the majority of VFs-positive isolates belonged to phylogenetic group B1 (Table 1A). There were no VFs-positive isolates classified into group B2 among beef cattle. Similarly, the analysis at the level of individuals revealed statistically significant differences (*p* = 0.04), in the beef cattle group 33.3% of animals carried *E. coli* positive for VFs, while among dairy cattle 14% of animals harboured *E. coli* positive for these genes. Significantly more animals living on the pasture (23.8%) than housed in the barn (8%) carried STEC isolates (*p* = 0.04) (Table 1B).

**Table 1:** Distribution of the profiles of virulence factors. (**A**) Among *E. coli* isolates in relation to the phylogenetic groups; results from the mixed logit model; (**B**) Among animals with *E. coli* positive for VFs profiles; results from the logistic model and tests of independence.

**(A)**										
**VFs Profiles**	**Number of VFs-Positive Isolates in the Phylogenetic Groups**									**OR (95% CI) ^a^**
	**Cattle Barn n = 147**					**Cattle Pasture n = 118**				
	**A**	**B1**		**B2**	**D**	**A**	**B1**	**B2**	**D**	
*escV*	1	2		-	-	-	-	-	-	-
*stx1*	-	1		-	-	1	1	-	-	1.63 (0.0035–762.296) ^b^
*stx2*	-	1		1	1	1	1	-	1	1.48 (0.293–7.512) ^b^
*ehxA*	-	-		-	1	1	-	-	-	1.50 (0.089–25.307) ^b^
*stx2*, *ehxA*	-	-		-	-	-	2	-	-	-
*stx2*, *estI*	-	-		-	-	4	-	-	-	-
*stx1*, *stx2*, *ehxA*	-	-		-	-	-	-	-	1	-
*hlyA*	-	-		-	-	-	8	-	1	-
*stx1*, *hlyA*	-	1		-	-	-	1	-	2	2.67 (0.214–33.283) ^b^
Total	1	5		1	2	7	13	-	5	
Total no. (%) of VFs-positive isolates	9 (6,1)					25 (21,2)				6.84 (1.036–45.220) *
No. (%) of STEC isolates *stx1* and/or *stx2*	5 (3.4)					15 (12.7)				6.18 (1.041–36.672) *
^a^ reference category—cattle barn; ^b^ the estimated OR, 95% CI refers to the sum of the VFs profiles of all the phylogroups; * statistically significant.										
**(B)**										
**VFs Profiles**	**Number of Animals with *E. coli* Positive for VFs Profiles**					**OR (95% CI) ^a^**				**Test of Independence *p*-value**
	**Cattle Barn n = 50**		**Cattle Pasture n = 42**							
*escV*	2		0			-				-
*stx1*	1		2			3.07 (0.266–35.491)				0.5622
*stx2*	3		3			1.54 (0.289–8.155)				0.680
*ehxA*	1		1			1.59 (0.096–26.539)				1
*stx2*, *ehxA*	0		1			-				-
*stx2*, *estI*	0		2			-				-
*stx1*, *stx2*, *ehxA*	0		1			-				-
*hlyA*	0		6			-				-
*stx1*, *hlyA*	1		2			2.45 (0.214–28.009)				0.590
Total no. (%) of animals with *E. coli* positive for VFs	7 (14)		14 (33.3)			3.07 (1.103–8.557) *				0.044 *
No. (%) of animals with STEC isolates *stx1* and/or *stx2*	4 (8)		10 (23.8)			3.59 (1.036–12.471) *				0.044 *
^a^ reference category—cattle barn; * statistically significant.										

**Figure 2:**
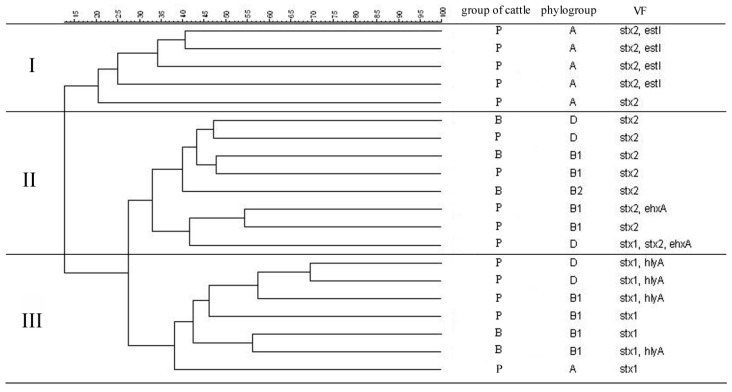
Dendrogram shows the similarity relationships between clustered STEC isolates based on BOX-PCR genomic patterns, phylogenetic grouping and virulence genes content using UPGMA grouping method. B-barn, P-pasture.

### 3.3. Similarity Analysis of STEC Isolates

The similarity of 20 *stx1* and/or *stx2* positive isolates were analysed based on the BOX-PCR genomic patterns, phylogenetic grouping and virulence genes content using UPGMA grouping method (Figure 2). The created dendrogram encompassed 20 STEC isolates divided into three clusters indicating significant diversity. The homology relations between the clusters were from 27% to 12%. Cluster I showed similarity from 40% to 20% and gathered isolates carrying *stx2* or *stx2* in combination with *estI*. Cluster II with similarity from 55% to 33% encompassed isolates carrying *stx2* or *stx2*, *ehxA* or *stx1*, *stx2*, *ehxA* profiles. Cluster III showed similarity from 70% to 38% and included isolates carrying *stx1* or *stx1*, *hlyA* profiles.

### 3.4. Antimicrobial Resistance 

The prevalence of resistance to each tested antimicrobial agents in both groups of animals is shown in Table 2A,B. *E. coli* isolates derived from dairy cattle revealed statistically significant higher resistance than isolates from beef cattle to ampicillin, cefuroxime, ceftazidime, neomycin, tetracycline and sulphamethoxazole. 

**Table 2:** Prevalence of resistance to 13 antimicrobial agents. (**A**) Among *E. coli* isolates from dairy and beef cattle; results from the mixed logit model; (**B**) Among animals with resistant *E. coli*; results from the logistic model and tests of independence.

**(A)**				
**Antimicrobial Agent**	**Number (%) of *E. coli* Isolates**			**OR (95% CI) ^a^**
	**Cattle Barn n = 147**		**Cattle Pasture n = 118**	
Ampicillin	31 (21,1)		8 (6,8)	0.21 (0.07–0.625) *
Cefuroxime	39 (26,5)		14 (11,9)	0.34 (0.157–0.733) *
Ceftazidim	11 (7.5)		1 (0.8)	0.12 (0.013–0.831) *
Streptomycin	30 (20,4)		14 (11,9)	0.35 (0.123–1.020)
Gentamicin	17 (11,6)		7 (5,9)	0.44 (0.148–1.330)
Neomycin	75 (51)		29 (24,6)	0.24 (0.116–0.51) *
Tetracycline	35 (23,8)		13 (11)	0.31 (0.103–0.938) *
Doxycycline	37 (25,2)		16 (13,6)	0.44 (0.184–1.027)
Sulphamethoxazole	19 (12,9)		6 (5,1)	0.36 (0.139–0.935) *
Trimethoprim	5 (3,4)		6 (5,1)	1.51 (0.441–5.190)
Chloramphenicol	8 (5,4)		11 (9,3)	1.79 (0.694–4.600)
Nalidixic acid	11 (7,5)		11 (9.3)	1.27 (0.531–3.044)
Norfloxacin	1 (0.7)		3 (2.5)	3.56 (0.251–50.652)
**Antimicrobial Susceptibility Characteristic**				
R (“1”)	121 (82.3)		69 (58.5)	0.28 (0.143–0.536) *
S (“0”)	26 (17.7)		49 (41.5)	
MDR (“1”)	25 (17)		10 (8.5)	0.39 (0.147–1.018)
NMDR (“0”)	122 (83)		108 (91.5)	
R—resistant to at least one agent; S—susceptible; MDR—multidrug-resistant; NMDR—nonmultidrug-resistant; ^a^ reference category—cattle barn; * statistically significant.				
**(B)**				
**Antimicrobial Agent**	**Number (%) of Animals with Resistant *E. coli***		**OR (95% CI) ^a^**	**Test of Independence *p*-value**
	**Cattle Barn n = 50**	**Cattle Pasture n = 42**		
Ampicillin	21 (42)	6 (14.3)	0.23 (0.0821–0.645) *	0.007 *
Cefuroxime	24 (48)	12 (28.6)	0.43 ( 0.182–1.034)	0.092
Ceftazidim	10 (20)	1 (2.4)	0.09 (0.012–0.797) *	0.010 *
Streptomycin	22 (44)	9 (21.4)	0.35 (0.138–0.875) *	0.039 *
Gentamicin	13 (26)	6 (14.3)	0.47 (0.163–1.384)	0.261
Neomycin	38 (76)	17 (40.5)	0.21 (0.088–0.525) *	0.001 *
Tetracycline	20 (40)	10 (23.8)	0.47 (0.189–1.162)	0.153
Doxycycline	23 (46)	13 (31)	0.53 (0.223–1.242)	0.208
Sulphamethoxazole	17 (34)	6 (14.3)	0.32 (0.114–0.919) *	0.033 *
Trimethoprim	5 (10)	6 (14.3)	1.50 (0.423–5.315)	0.540
Chlo­ramphenicol	8 (16)	9 (21.4)	1.43 (0.498–4.116)	0.690
Nalidixic acid	10 (20)	9 (21.4)	1.09 (0.397–3.000)	1
Norfloxacin	1 (2)	2 (4.8)	2.45 (0.214–28.008)	0.590
^a^ reference category—cattle barn; * statistically significant.				

*E. coli* isolated from dairy cows demonstrated the highest resistance rate for neomycin (51%) and the lowest for norfloxacin (0.7%). The highest resistance rate was observed for neomycin (24.6%) among isolates derived from beef cattle. These isolates showed the lowest resistance for ceftazidime (0.8%). In the group of dairy cattle 82.3% of isolates were resistant to at least one agent, whereas 17% were multidrug-resistant. Among isolates from beef cattle 58.5% were resistant to at least one agent, while 8.5% were multidrug-resistant (Table 2A). The results revealed statistically significant differences in the distribution of isolates resistant to at least one agent between two examined groups of the cattle. The analysis at the level of individuals (Table 2B) has shown that statistically significant more animals from the barn than from the pasture carried *E. coli* resistant to ampicillin, ceftazidime, streptomycin, neomycin and sulphamethoxazole.

### 3.5. Frequency of Antimicrobial Resistance Genes

Isolates were screened for the presence of resistance genes according to their antimicrobial susceptibility profile. Resistance to ampicillin, cephalosporins, streptomycin, tetracycline, sulphamethoxazole and trimethoprim among *E. coli* isolates derived from cattle has been reported [20] and it is important considering the possibility of transmission to humans, thus genetic determinants associated with such a resistance were tested in this study. A summary of the frequency of detected resistance genes was presented in Table 3. Determinants associated with resistance to cephalosporins *bla*_CTX-M_, sulfamethoxazole *sul1*, *sul2* and trimethoprim *dfrA*, *dfrA7/dfrA17* were not detected. None of the *E. coli* isolates derived from the cattle living on the pasture were positive for tested resistance genes.

**Table 3:** Prevalence of the antimicrobial resistance genes among phenotypically resistant *E. coli* and among animals with resistant *E. coli* from dairy cattle.

Antimicrobial Agent	Resistance Gene	Number (%) of Isolates with Resistance Genes	Number (%) of Animals with *E. coli* Positive for Resistance Genes
		Cattle Barn	
Ampicillin		n = 31	n = 21
	*bla*_TEM_	6 (19.4)	4 (19)
	*bla*_SHV_	4 (12.9)	3 (14.3)
Streptomycin		n = 30	n = 22
	*aadA1*	10 (33.3)	6 (27.3)
Tetracycline		n = 35	n = 20
	*tetA*	9 (25.7)	5 (25)
	*tetB*	1 (2.9)	1 (5)
	*tetC*	1 (2.9)	1 (5)

### 3.6. Associations between Virulence Factors and Antimicrobial Resistance 

The results revealed that there was no significant association (*p* > 0.05) between VFs and antimicrobial resistance, VFs-positive isolates were uniformly distributed among resistant and susceptible isolates.

## 4. Discussion

Monitoring of genetic diversity of commensal *E. coli* derived from farm animals is important for many reasons. The ability of *E. coli* to the acquisition of many different virulence factors can result in the emergence of aggressive strains, particularly dangerous to human health, as in the case of *E. coli* O104:H4 outbreak [21]. Moreover, last years, many publications provided data concerning the increasing antimicrobial resistance among *E. coli* isolated from food-producing animals. Commensal *E. coli* inhabiting the gastrointestinal tract of farm animals or present on animal products are regarded as potential reservoir of resistance genes. If the infections are caused by strains harbouring combination of virulence factors and antimicrobial resistance determinants this may pose particularly serious problem to human health and medicine. 

This study demonstrated significant differences between distribution of phylogroups among *E. coli* isolates from two tested groups of animals. Phylogroup B1 predominated among isolates from beef cattle, such results are consistent with previous reports [22,23], whereas *E. coli* isolates from dairy cattle were distributed almost uniformly to phylogroups A, B1, D. Phylogroup B2 was the least numerous, both in the case of dairy and beef cattle. These results confirm the previous observations which revealed that the diet of the host could be a factor affecting the phylogenetic structure of *E. coli* [23,24]. The diet of beef cattle consisted of unprocessed, natural for ruminant foods (grass and straw) promoted the predomination of phylogroup B1, while partially processed foods served to dairy cattle in the barn caused the shift in the distribution of the phylogenetic groups, where B1 isolates became much less common. In the wild herbivorous hosts the dominance of group B1 in the phylogenetic structure of commensal *E. coli* had been described previously [23,25,26]. The diet did not affect the prevalence of the phylogroup B2.

The identification of virulence factors showed that cattle were the reservoir of VFs typical for intestinal and extraintestinal pathotypes and that prevalence of VF-positive *E. coli* isolates and animals with such isolates was significantly higher among beef cattle than the dairy cows. The previous report with the same panel of identified VFs [22] indicated similar prevalence rates of VFs-positive isolates among *E. coli* isolates from beef cattle and dairy cattle. In the present study similar percentage of VFs-positive isolates were identified among beef cattle but in contrast significantly less isolates derived from dairy cows carried VFs. The comparison of frequency of STEC isolates and animals with STEC isolates also showed significantly higher prevalence of such *E. coli* among beef cattle than in dairy cows. Uno *et al.* [22] reported similar rates of prevalence of STEC isolates among beef cattle, but in dairy cattle STEC were not detected. Some earlier studies reported wide range of STEC prevalence in cattle faeces [27,28]. The present study suggests the higher probability of infection with STEC pathotypes among cattle grazing on the pasture, which is consistent with earlier reports [27].

The analysis of the dendrogram presenting the similarity relationships between 20 STEC isolates revealed the occurrence of significant genetic diversity of these isolates, excluding the possibility of their transmission between individual animals and indicating horizontal transfer of the genetic elements harbouring *stx1* and *stx2* genes. In *E. coli* lysogenic for Shiga toxin-converting bacteriophages, production of Shiga toxins must be preceded by prophage induction. Recently the hypothesis concerning altruism of STEC isolates has been proposed [29,30]. *E. coli* isolates may be attacked by unicellular predator like *Tetrahymena*. The predator produces hydrogen peroxide which may induce Shiga toxin-converting prophages in a small fraction of STEC isolates, resulting in the production of Shiga toxin in amounts enough to kill the predator. This way a small fraction of STEC isolates can protect another *E. coli* isolates before the attack of the predator, so from evolutionary point of view it is advantageous to carry genes encoding Shiga toxin. The results in the present study are consistent with the presented approach, the prevalence of STEC among *E. coli* isolates from pasture is significantly higher, but in this habitat the probability of attack from unicellular predator is obvious. The significant genetic diversity of STEC isolates based on BOX-PCR genomic patterns and phylogenetic grouping also indicate that bacteriophages propagation among many different strains is favoured. 

The presented results suggest that the habitat and environmental factors may affect the prevalence and complexity of VFs-positive isolates. Limited space in the barn environment and anthropogenic pressure generates a decrease in VFs prevalence and simplification of virulence profiles, while the open pasture environment provides the opportunity to spread VFs-positive *E. coli* isolates.

This study revealed significant differences in antimicrobial resistance prevalence of *E.coli* between two analysed groups of cattle. This result was confirmed at the level of the sets of *E. coli* isolates as well as at the level of the individual animals carrying resistant *E. coli* in analysed groups of the cattle. Isolates from dairy cows showed higher resistance to 11 out of 13 tested antimicrobial agents, at a statistically significant level for six agents. It was reported [31,32] that dairy cattle usually carried more antimicrobial resistant enteric bacteria than beef cattle, due to the fact that dairies used more antimicrobials, particularly to treat mastitis. Some studies have shown that application of antimicrobial agents causes the increase in the level of resistance as a consequence of selective pressures exerted during animal production [33]. In general the present results indicated a high prevalence of resistance among *E. coli* isolates from analysed cattle, with resistance rates to at least one agent 82.3% and 58.5% among dairy and beef cattle respectively. The multidrug resistance was significantly lower 17% and 8.5% among dairy and beef cattle respectively. It is in agreement with previous reports [34,35], however, some other reports indicated much lower rates of resistance among *E. coli* isolates [6,36]. Some reports indicated that prevalence of resistance not necessarily was the direct result of selection pressure by using antimicrobial agents in breeding [37,38]. Call *et al.* [31] proposed the model that illustrated a possible emergence of the genetic linkage between resistance genes and genes responsible for colonization of intestinal tract of the cattle. This linkage allows to maintain the resistant bacteria within the host animals even without antimicrobial selection pressure.

The selected genetic determinants associated with resistance to ampicillin, streptomycin and tetracycline were present among resistant *E. coli* isolates derived from dairy cattle, the remaining resistant isolates may have carried one of the other resistance determinant. None of the tested resistance genes were found among resistant isolates from beef cattle, it may be the result of much lower level of resistance in this group of isolates and the presence of other resistance determinants than tested. 

The results of these studies did not show any association between the presence of tested VFs and antimicrobial resistance among *E. coli* isolates. Few previous studies revealed that bovine STEC isolates from healthy cattle did not constitute a high risk of spreading antibiotic-resistant *E. coli* to humans [35,39]. 

Some previous studies reported that antibiotics usage may strongly influence the phylogenetic group distribution of *E. coli* isolates [40]. Bukh *et al.* [41] reported that resistance to multiple antibiotics was mostly prevalent for phylogroups A and D, and decreased in the order of group D > A > B1 > B2. Therefore isolates classified to B1 phylogroup were less resistant than to groups A and D. Our results confirmed this observation, because the lower frequency of phylogroup B1 in *E. coli* from dairy cattle might be a consequence of earlier selection pressure by the use of antimicrobial agents. The phylogroup B1 predominated among isolates derived from beef cattle, but the level of antimicrobial resistance was significantly lower than among isolates from dairy cattle. It was also previously reported that the most of isolates obtained from beef cattle and dairy cattle carrying virulence genes were found in phylogenetic group B1 [22,42,43]. The present results have shown that in both groups of cattle the majority of VFs-positive isolates belonged to phylogenetic group B1, which suggests that the lower prevalence of VFs-positive isolates among *E. coli* from dairy cattle was the consequence of a lower prevalence of phylogroup B1. Moreover, there were no associations between the prevalence of VFs and antimicrobial resistance, which suggested that the lower prevalence of VFs-positive isolates in dairy cattle, might be partially the result of the elimination of sensitive VFs-positive isolates through earlier antimicrobial pressure.

## 5. Conclusions 

The study encompassed the analysis of the phylogenetic structure, the distribution of virulence factors and prevalence of antimicrobial resistance among *E. coli* isolated from healthy dairy cattle housing in the barn and beef cattle living on the ecological pasture. Both statistical approaches revealed the similar picture indicating the significant differences between frequencies of VFs and antimicrobial resistance among the cattle in two different habitats. The results suggest the associations between the farm environment and the gene content of commensal *E. coli*. These findings may indicate the existence of the relationships and the influence of habitat and diet on the genetic diversity of commensal *E. coli* isolates derived from the cattle. The results have shown that *E. coli* isolates with higher level of antimicrobial resistance are more prevalent in the conventional barn habitat, whereas the ecological pasture habitat promotes the spreading of the VFs-positive isolates. The genome dynamics of *E. coli* is important in infectious disease processes. The genome alterations lead to rapid adaptation of *E. coli* to new ecological niches and may affect disease severity. Therefore, further research will allow a better understanding of the farm environment role in the transmission of virulence factors and antimicrobial resistance.
